# Paclitaxel-Induced Apoptosis Is BAK-Dependent, but BAX and BIM-Independent in Breast Tumor

**DOI:** 10.1371/journal.pone.0060685

**Published:** 2013-04-05

**Authors:** Anna V. Miller, Mark A. Hicks, Wataru Nakajima, Amanda C. Richardson, Jolene J. Windle, Hisashi Harada

**Affiliations:** 1 Department of Oral and Craniofacial Molecular Biology, School of Dentistry, Massey Cancer Center, Virginia Commonwealth University, Richmond, Virginia, United States of America; 2 Department of Pathology, School of Medicine, Virginia Commonwealth University, Richmond, Virginia, United States of America; 3 Department of Human and Molecular Genetics, School of Medicine, Massey Cancer Center, Virginia Commonwealth University, Richmond, Virginia, United States of America; University of Manitoba, Canada

## Abstract

Paclitaxel (Taxol)-induced cell death requires the intrinsic cell death pathway, but the specific participants and the precise mechanisms are poorly understood. Previous studies indicate that a BH3-only protein BIM (BCL-2 Interacting Mediator of cell death) plays a role in paclitaxel-induced apoptosis. We show here that BIM is dispensable in apoptosis with paclitaxel treatment using *bim^−/−^* MEFs (mouse embryonic fibroblasts), the *bim^−/−^* mouse breast tumor model, and shRNA-mediated down-regulation of BIM in human breast cancer cells. In contrast, both *bak* ^−/−^ MEFs and human breast cancer cells in which BAK was down-regulated by shRNA were more resistant to paclitaxel. However, paclitaxel sensitivity was not affected in *bax^−/−^* MEFs or in human breast cancer cells in which BAX was down-regulated, suggesting that paclitaxel-induced apoptosis is BAK-dependent, but BAX-independent. In human breast cancer cells, paclitaxel treatment resulted in MCL-1 degradation which was prevented by a proteasome inhibitor, MG132. A Cdk inhibitor, roscovitine, blocked paclitaxel-induced MCL-1 degradation and apoptosis, suggesting that Cdk activation at mitotic arrest could induce subsequent MCL-1 degradation in a proteasome-dependent manner. BAK was associated with MCL-1 in untreated cells and became activated in concert with loss of MCL-1 expression and its release from the complex. Our data suggest that BAK is the mediator of paclitaxel-induced apoptosis and could be an alternative target for overcoming paclitaxel resistance.

## Introduction

Breast cancer is a leading cause of death among women. Understanding breast cancer at the molecular level is imperative for finding more effective approaches to successfully treat these patients. Microtubule inhibitors are among the most frequently used agents for breast cancer treatment, with proven efficacy in both localized and metastatic disease. Paclitaxel (Taxol) is a member of the taxane class of anti-neoplastic microtubule damaging agents and exhibits activity against a wide range of human malignancies including breast cancer [1], [2]. Paclitaxel stabilizes microtubules, resulting in G2/M cell cycle arrest, and continuous treatment with paclitaxel ultimately leads to cell death. However, the precise mechanisms of how this mitotic arrest triggers cell death are still unclear.

When cells undergo paclitaxel-induced cell death, the BCL-2 family-dependent mitochondrial apoptotic pathway is activated [3], [4]. The BCL-2 family is subdivided into three main groups based on regions of BCL-2 homology (BH) and function: multi-domain anti-apoptotic (BCL-2, MCL-1, BCL-X_L_), multidomain pro-apoptotic (BAX, BAK), and BH3-only pro-apoptotic (for example, BIM, BID, BAD, PUMA). The BH3-only proteins clearly act upstream of BAX and BAK because they cannot induce apoptosis in cells lacking both BAX and BAK. BH3-only proteins cause cytochrome c release by activating BAX and/or BAK, and the anti-apoptotic BCL-2 family of proteins prevents this process [3], [4]. Among the BCL-2 family cell death regulators, a BH3-only protein BIM (Bcl-2 Interacting Mediator of cell death) has been shown to play a role in paclitaxel-induced cell death. Down regulation of BIM by siRNA delays paclitaxel-mediated apoptosis in cell based models [5], [6], [7], [8]. In addition, E1A and dominant-negative p53 transformed BMK (baby mouse kidney) cell lines of *bim^−/−^* mice showed the importance of BIM expression for paclitaxel cytotoxicity [9]. On the contrary, shRNA-mediated BIM depletion studies demonstrate that BIM is not required for paclitaxel cytotoxicity in breast cancer cell lines [10].

It is imperative to define the contribution of BIM in paclitaxel-induced apoptosis in order to rationally develop enhanced treatment strategies. Although cell culture model systems are well-suited for biochemical questions, they are relatively contrived with regard to factors such as substrate attachment and growth factor availability, both of which have profound effects on cellular susceptibility to apoptosis. For this reason, it is important to extend the knowledge gained from cell culture settings to *in vivo* models that more closely mimic the cell type, cellular environment, and tumor evolution processes encountered in human tumors. Thus, we obtained the MMTV**-** *ErbB2* line of mice, a well-established breast cancer mouse model, and generated a breeding colony of MMTV-*ErbB2*/*bim* ^−/−^ mice. In addition to this mouse tumor model, all of our *in vitro* and *in vivo* models support that BIM is dispensable in paclitaxel-induced apoptosis. Furthermore, both *bak* ^−/−^ mouse embryonic fibroblasts (MEFs) and human breast cancer cells in which BAK is down-regulated by shRNA are more resistant to low doses of paclitaxel treatment. Our data suggest that BAK is a critical mediator of paclitaxel-induced apoptosis through its release from the complex with MCL-1.

## Materials and Methods

### Cell Lines and Culture

Simian virus 40 (SV40)-immortalized MEFs were kindly provided by Dr. Stanley Korsmeyer (Dana-Farber Cancer Institute, Boston, MA) [11], [12]. All human breast cancer cells were purchased from the American Tissue Culture Collection (Manassas, VA). Cells were cultured in DMEM supplemented with 10% heat-inactivated fetal bovine serum (FBS) and penicillin G/streptomycin at 37°C in a humidified, 5% CO_2_ incubator.

### Mice

The mice study was carried out in strict accordance with the recommendations in the Guide for the Care and Use of Laboratory Animals of the National Institutes of Health. The protocol was approved by the Institutional Animal Care and Use Committee of Virginia Commonwealth University [Protocol Number: AD20132 (PI: Hisashi Harada)]. MMTV-*ErbB2* mice [13] were purchased from The Jackson Laboratory (Bar Harbor, ME). *Bim* ^−/−^ mice [12] were obtained from Dr. Stanley Korsmeyer (Dana-Farber Cancer Institute, Boston, MA). The mice were interbred to generate cohorts of MMTV-*ErbB2/bim^+/+^* and MMTV-*ErbB2/bim^−/−^* female mice. The *bim* ^−/−^ mice were originally obtained in a C57BL/6 background, while the MMTV-*ErbB2* mice were obtained in an FVB background. However, they were subsequently maintained in our laboratory in a mixed genetic background including C57BL/6 and FVB. The presence or absence of *ErbB2* and *bim* alleles in offspring of the interbreedings was determined by PCR. Genomic DNA was extracted from a small piece of tail cut from each animal at the time of weaning. PCR reactions were carried out as described previously [12]. In MMTV-*ErbB2* transgenic mice, mammary tumors arise primarily in females, and the kinetics of tumor onset is significantly accelerated by pregnancy and lactation. To avoid the complicating effects of pregnancy on tumorigenesis, we maintained all experimental females as virgins. Tumor volume in mm^3^ was estimated using the formula: vol = (L x W^2^)/2, where L is the larger dimension and W is the smaller dimension in millimeter with caliper measurement. Tumor growth was monitored from the time of initial tumor detection until the tumor reached a size of ∼500 mm^3^. The mice then began receiving paclitaxel treatment (15 mg/kg) by intra-peritoneal injection daily for 9 days [14]. Tumor size was measured every other day to determine tumor growth response to paclitaxel. The measurement used in the analysis was based on the area under the tumor growth curve (AUC). The AUC is determined by numerical methods for each mouse, and is then normalized over time to give an AUC/day, which is referred to as the mean growth rate (MGR). The MGR values for all mice in a treatment group were averaged to provide the mean MGR for that group, along with the standard error of the mean. Tumor growth was also measured in vehicle-treated mice per genotype for 9 days after their tumor reached a size of ∼500 mm^3^.

### Culture of Primary Mouse Mammary Epithelial Cells

The protocol was approved by the Institutional Animal Care and Use Committee of Virginia Commonwealth University [Protocol Number: AD20132 (PI: Hisashi Harada)]. A size of ∼500 mm^3^ tumors was mechanically processed, dissociated in collagenase/dispase (Roche, Indianapolis, IN) for 2 hours at 37°C, washed with PBS +1 mM EDTA and cultured in DMEM supplemented with 2% FBS and Mammary Epithelial Growth Supplement (MEGS) (Invitrogen, Carlsbad, CA) at 37°C in a humidified, 5% CO_2_ incubator.

### Chemicals and Antibodies

Paclitaxel was purchased from Sigma (St. Louis, MO) for cell culture studies and from SAGENT Pharmaceuticals (Schaumburg, IL) for the animal study. Roscovitine and MG132 were purchased from Sigma and Millipore (Billerica, MA), respectively. Antibodies were purchased as follows: BIM, BCL-X_L_, Cleaved PARP, Cleaved Caspase-3, and ErbB2 from Cell Signaling Technology (Beverly, MA); mouse MCL-1 from Rockland Immunochemicals (Gilbertsville, PA); human MCL-1 from Enzo Life Sciences (Farmingdale, NY); BAX, Cyclin B1, Histone H1, α-Tubulin, and normal rabbit IgG from Santa Cruz Biotechnology (Santa Cruz, CA); BAK (06–536), BAK (Ab-1), and phospho-Histone H1 from Millipore.

### Plasmid Transfection and Lentivirus Infection

The lentiviral short-hairpin RNA (shRNA)-expressing constructs were purchased from Open Biosystems (Huntsville, AL). The constructs were transfected into 293T packaging cells along with the packaging plasmids, and the lentivirus-containing supernatants were used to transduce human breast cancer cells.

### Cell Death Assay

Cell death was quantified by the amount of DNA fragmentation using the Cell Death Detection ELISA^plus^ kit (Roche) according to the manufacturer’s protocol, or the cell numbers determined by trypan-blue exclusion.

### Immunoprecipitation and Western Blot Analyses

Whole cell lysates were prepared with CHAPS lysis buffer [20 mM Tris (pH 7.4), 137 mM NaCl, 1 mM dithiothreitol (DTT), 1% CHAPS (3-[(3-Cholamidopropyl)dimethylammonio]-1-propanesulfonate), a protease inhibitor cocktail, and phosphatase inhibitor cocktails (Sigma)]. For immunoprecipitation, equal amounts of protein were precleared with protein A/G beads (Pierce, Rockford, IL), and incubated with the appropriate antibodies on ice for 2 hours. Then the antibody complexes were captured with protein A/G beads at 4°C for 1 hour. After washing three times with the same lysis buffer, the beads were re-suspended in the sample buffer and separated by SDS-PAGE. For Western blot analyses, equal amounts of proteins were loaded on a SDS-acrylamide gel, transferred to a nitrocellulose membrane and analyzed by immunoblotting. Phosphorylated MCL-1 was detected on 10% SDS-PAGE with 25 µM Phos-tag acrylamide (Wako, Richmond, VA) according to the manufacturer’s protocol [15].

### Statistical Analysis

Values represent the means ± SD for three separate experiments. The significance of differences between experimental variables was determined using the Student’s t test. Values were considered statistically significant at *P*<0.05.

## Results

### BIM is Dispensable for Paclitaxel-induced Apoptosis in both *in vitro* and *in vivo*

**Figure 1:**
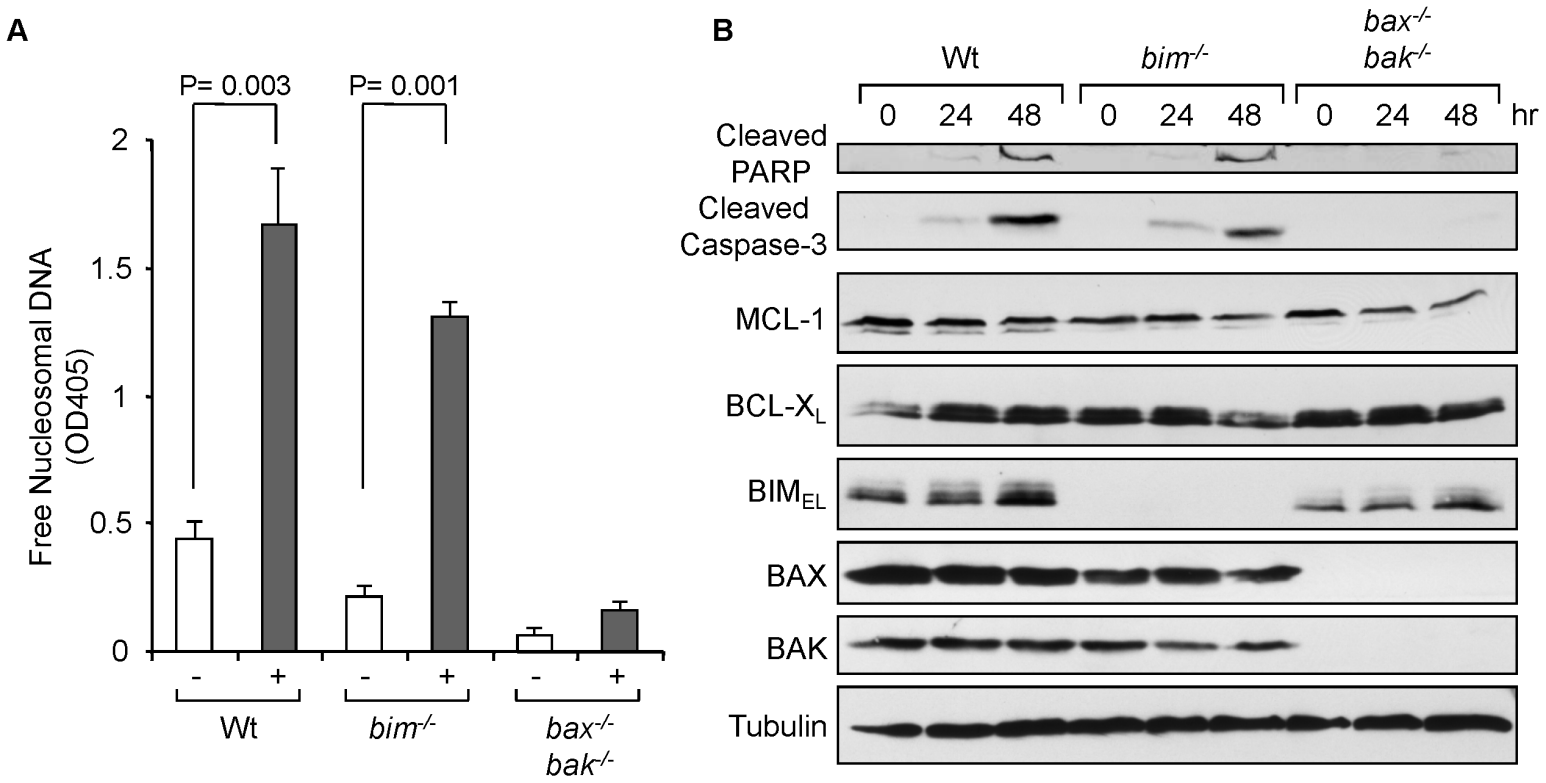
BIM is dispensable for paclitaxel-induced apoptosis in MEFs. (A) MEFs were treated with 50 nM of paclitaxel for 48 hrs. Cell death was determined by DNA fragmentation using the Cell Death Detection ELISA^plus^ kit (Roche). Average values from triplicate samples are shown as representative of two independent experiments. (B) MEFs were treated with 50 nM of paclitaxel for the indicated times. Total cell extracts were subjected to Western blotting with the indicated antibodies.

In order to examine the role of BIM in paclitaxel-induced cell death, we prepared mouse embryonic fibroblasts (MEFs) from wild-type and *bim^−/−^* mice and treated with 20 nM of paclitaxel for 48 hrs. Free nucleosomal DNA, indicative of DNA cleavage followed by apoptotic cell death, was observed in both wild-type and *bim^−/−^* MEFs (Figure 1A). Cleaved-PARP was also detected at 24 hours after the treatment and increased at 48 hours in both wild-type and *bim^−/−^* MEFs (Figure 1B). In contrast, both DNA cleavage and cleaved PARP was undetectable in *bax^−/−^bak^−/−^* (dKO) MEFs, consistent with the previous observations. BIM was induced over the time period in both wild-type and dKO MEFs, suggesting that BIM may receive a signal(s) of mitotic checkpoint. These results suggest that paclitaxel-induced cell death depends on BAX and/or BAK mediated mitochondrial apoptotic pathway, but is independent of BIM.

**Figure 2:**
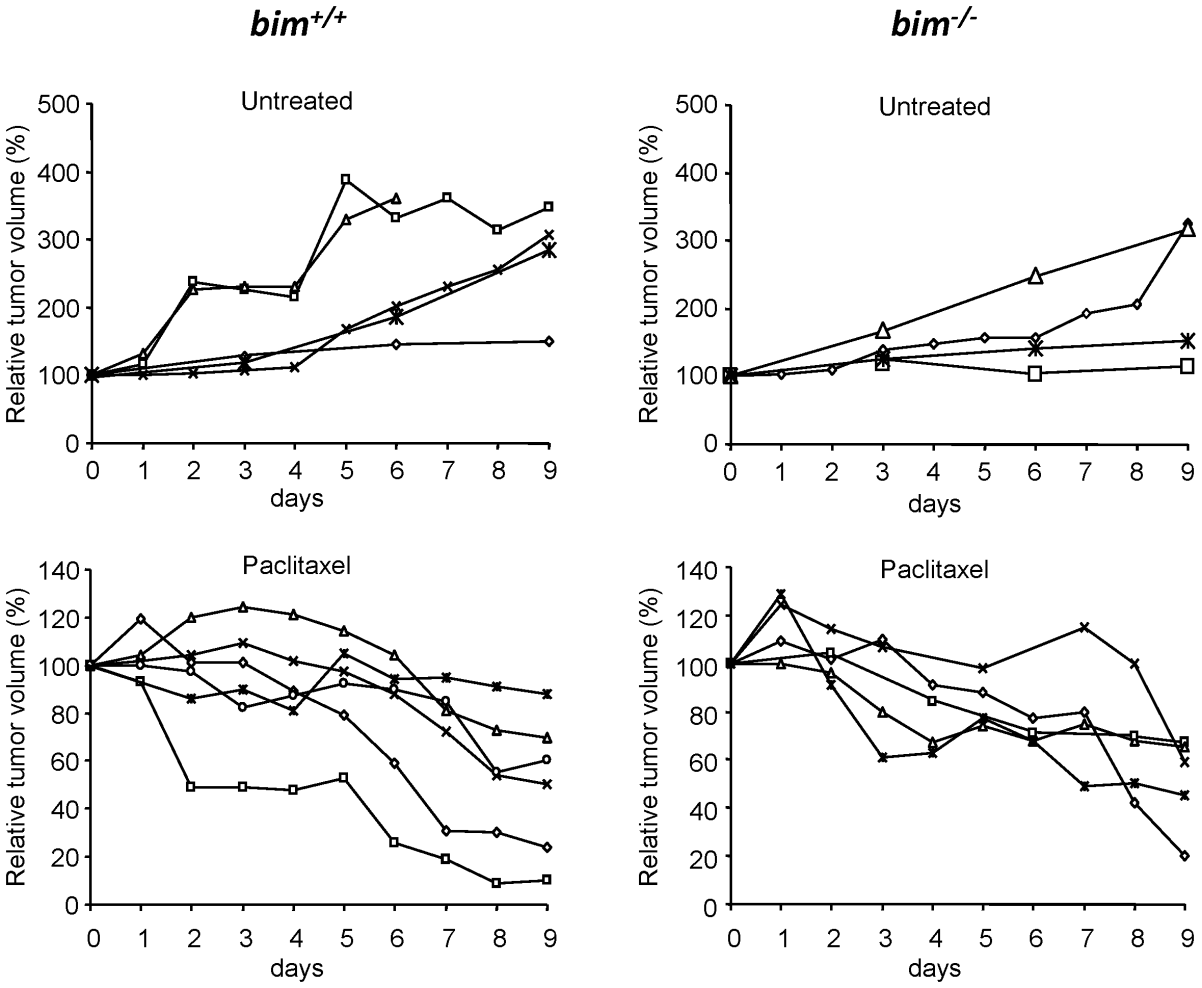
Mammary tumor growth response to paclitaxel in MMTV-*ErbB2/Bim^+/+^* and MMTV-*ErbB2/Bim^−/−^* mice. Tumor growth curves for untreated and treated mammary tumors arising MMTV-*ErbB2/Bim^+/+^* and MMTV-*ErbB2/Bim^−/−^* mice are shown. Tumor-bearing mice were treated nine consecutive days with paclitaxel (15 mg/kg) and tumor growth was monitored by caliper measurements. Each line represents the growth of an individual tumor.

**Figure 3:**
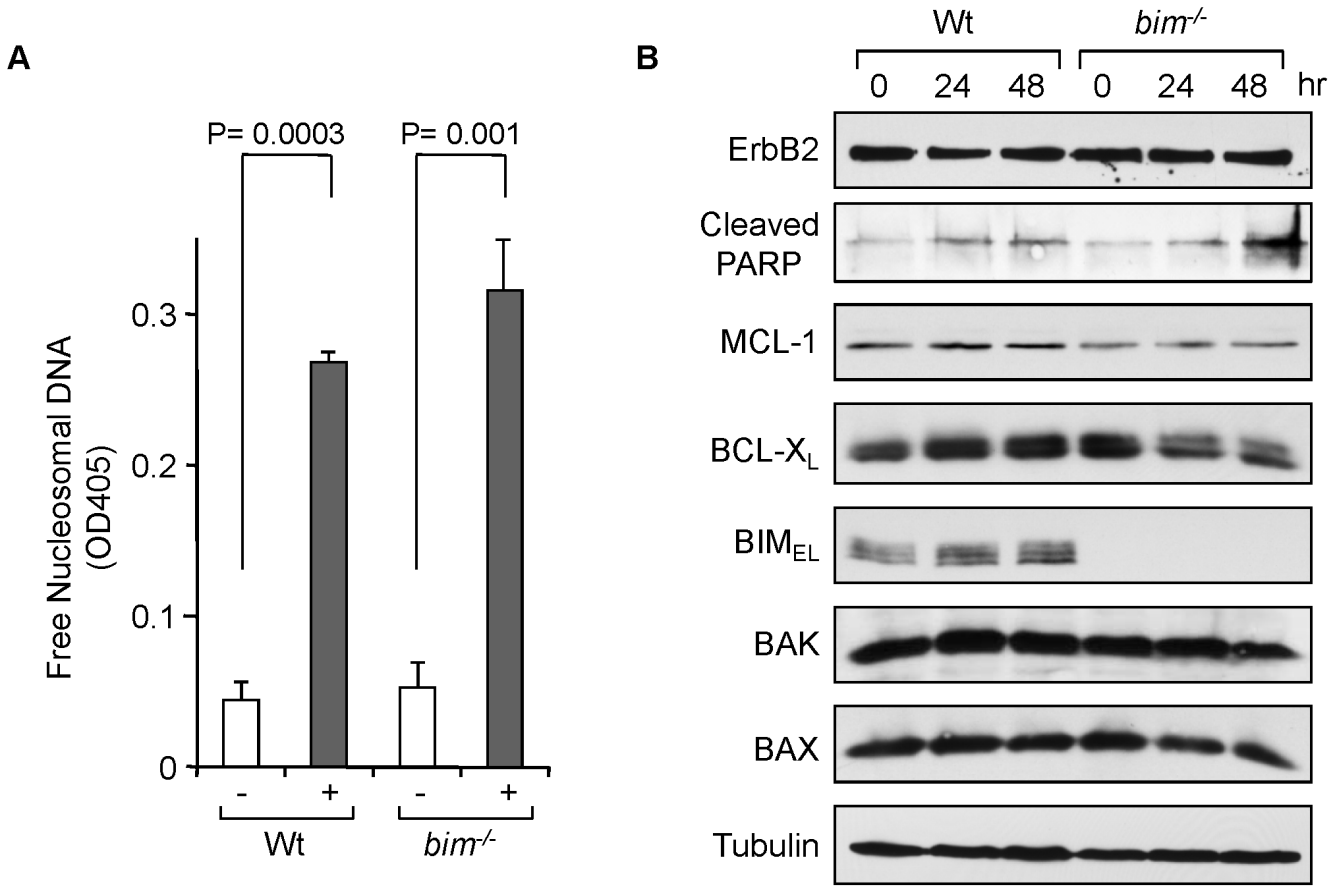
BIM is dispensable for paclitaxel-induced apoptosis in *ex vivo* mouse mammary tumors. (A) Mouse mammary epithelial cells were treated with 1 µM of paclitaxel for 48 hrs. Cell death was determined by DNA fragmentation using the Cell Death Detection ELISA^plus^ kit (Roche). Average values from triplicate samples are shown as representative of two independent experiments. (B) Mouse mammary epithelial cells were treated with 1 µM of paclitaxel for the indicated time. Total cell extracts were subjected to Western blotting with the indicated antibodies.

To further investigate whether paclitaxel could induce BIM-independent cell death, we established an *in vivo bim^−/−^* mouse breast tumor model. *ErbB2* (*HER2, neu*) is a proto-oncogene that belongs to the epidermal growth factor receptor (EGFR) family [16], [17]. ErbB2 is frequently (∼30%) overexpressed in breast tumors [18]. MMTV-ErbB2 transgenic mice produce spontaneous breast tumors within 6–8 months of average tumor onset [13]. To generate MMTV-*ErbB2/bim^−/−^* mice, *bim* ^−/−^ mice were interbred to MMTV-*ErbB2* transgenic mice. The MMTV-*ErbB2* female mice with *bim^+/+^, bim^+/−^, and bim^−/−^* were born with the numbers of 86∶76:28, suggesting that BIM contributes to the development of mice. However, tumor onset was 6–8 months in average regardless of the *bim* status. As we previously established the treatment protocol [14], tumor-bearing mice were treated with 15 mg/kg of paclitaxel for 9 consecutive days. Without the treatment, tumors in both *bim^+/+^* and *bim^−/−^* mice grew with MGR = 23.5%/day for *bim^+/+^* and MGR = 12.0%/day for *bim^−/−^*, respectively, suggesting that BIM plays a role in ErbB2-mediated tumor growth. With paclitaxel treatment, tumors in both genotypes shrink in similar ratio (MGR = −5.2%/day) (Figure 2). We further confirmed BIM-independent cell death induced by paclitaxel in breast tumors *ex vivo*. Treatment with paclitaxel in mouse mammary epithelial cells from both *bim^+/+^* and *bim^−/−^* induced apoptosis to a similar extent (Figure 3A). BIM was clearly induced in *bim^+/+^* cells as similarly observed in MEFs, suggesting that a signal(s) of mitotic checkpoint was transduced (Figure 3B). Taken together, all experiments in *in vitro, in vivo* and *ex vivo* indicate that BIM is dispensable in paclitaxel-induced apoptosis.

### Paclitaxel-induced Apoptosis is BAK-dependent

**Figure 4:**
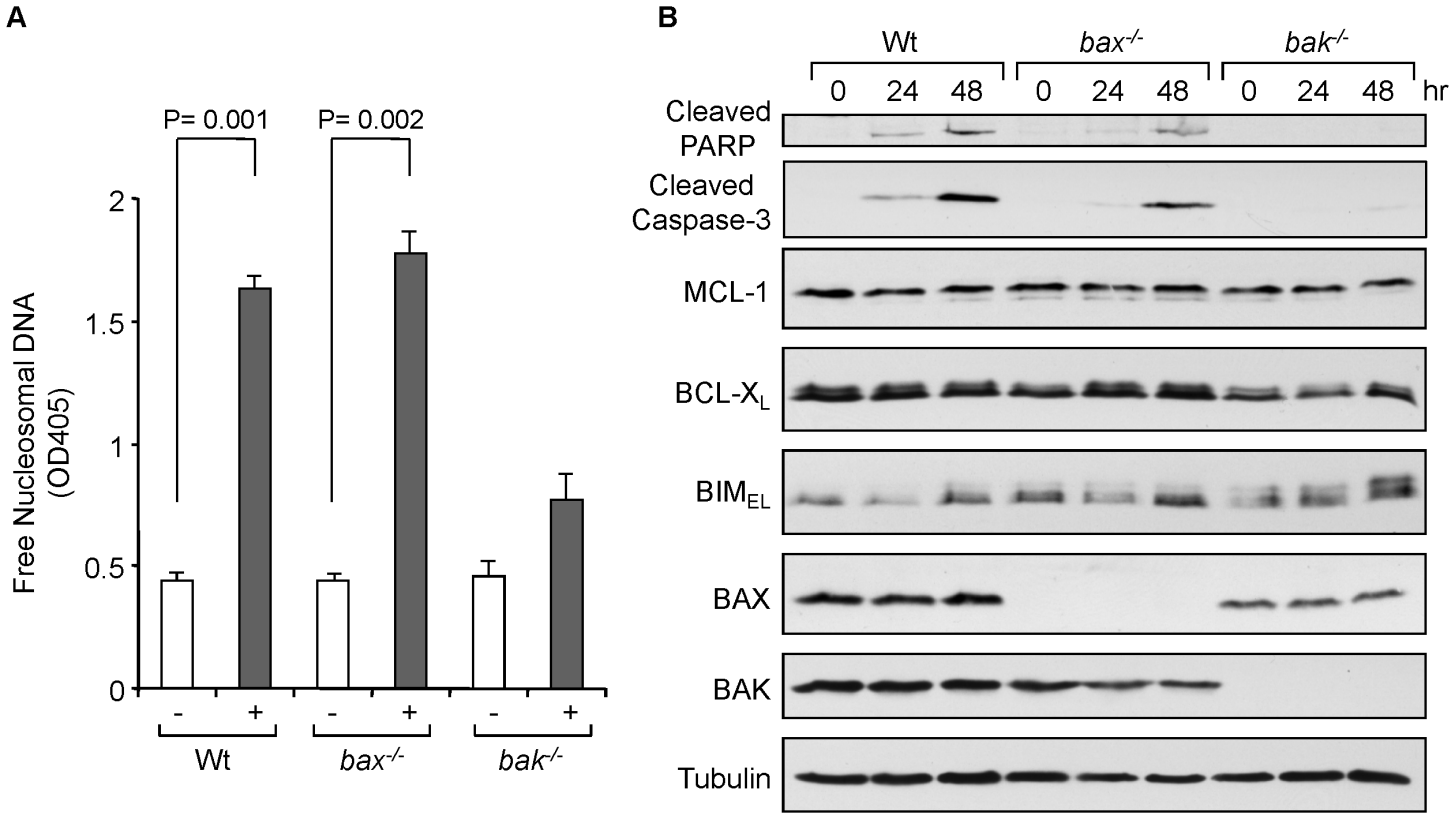
BAK plays a role in paclitaxel-induced apoptosis in MEFs. (A) MEFs were treated with 50 nM of paclitaxel for 48 hrs. Cell death was determined by DNA fragmentation using the Cell Death Detection ELISA^plus^ kit. Average values from triplicate samples are shown as representative of two independent experiments. (B) MEFs were treated with 50 nM of paclitaxel for the indicated times. Total cell extracts were subjected to Western blotting with the indicated antibodies.

Since *bax^−/−^bak^−/−^* MEFs were resistant to paclitaxel-induced cell death (Figure 1), we next examined whether BAX and/or BAK were required for the cell death. We took advantage of using *bax^−/−^* and *bak^−/−^* MEFs with paclitaxel treatment. *Bax^−/−^* MEFs showed similar amounts of apoptosis with wild-type MEFs, judged by DNA fragmentation and PARP cleavage (Figure 4A and 4B). In contrast, *bak^−/−^* MEFs were resistant to apoptosis induced by paclitaxel treatment to a similar extent as dKO MEFs. These results suggest that BAK plays a crucial role in paclitaxel-induced apoptosis and BAX is redundant.

**Figure 5:**
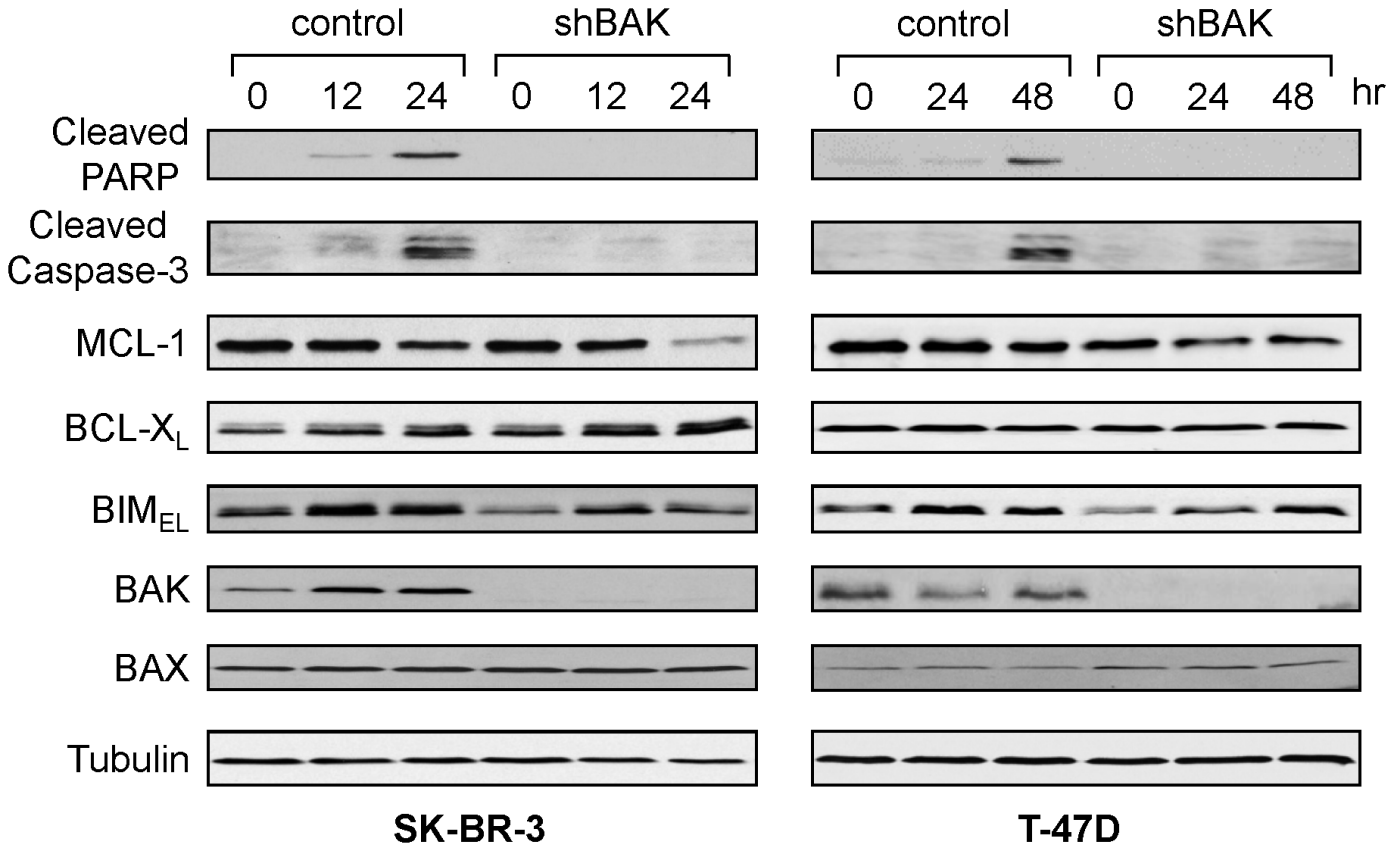
BAK plays a role in paclitaxel-induced apoptosis in human breast cancer cells. SK-BR-3 and T47D cells were infected with lentiviruses expressing shRNAs for non-targeting control or BAK. Puromycin-resistant cells were pooled after each infection. Cells were treated with 20 nM paclitaxel for SK-BR-3 or 50 nM for T47-D for the indicated times and equal amounts of total cell extracts were subjected to Western blotting with the indicated antibodies.

**Figure 6:**
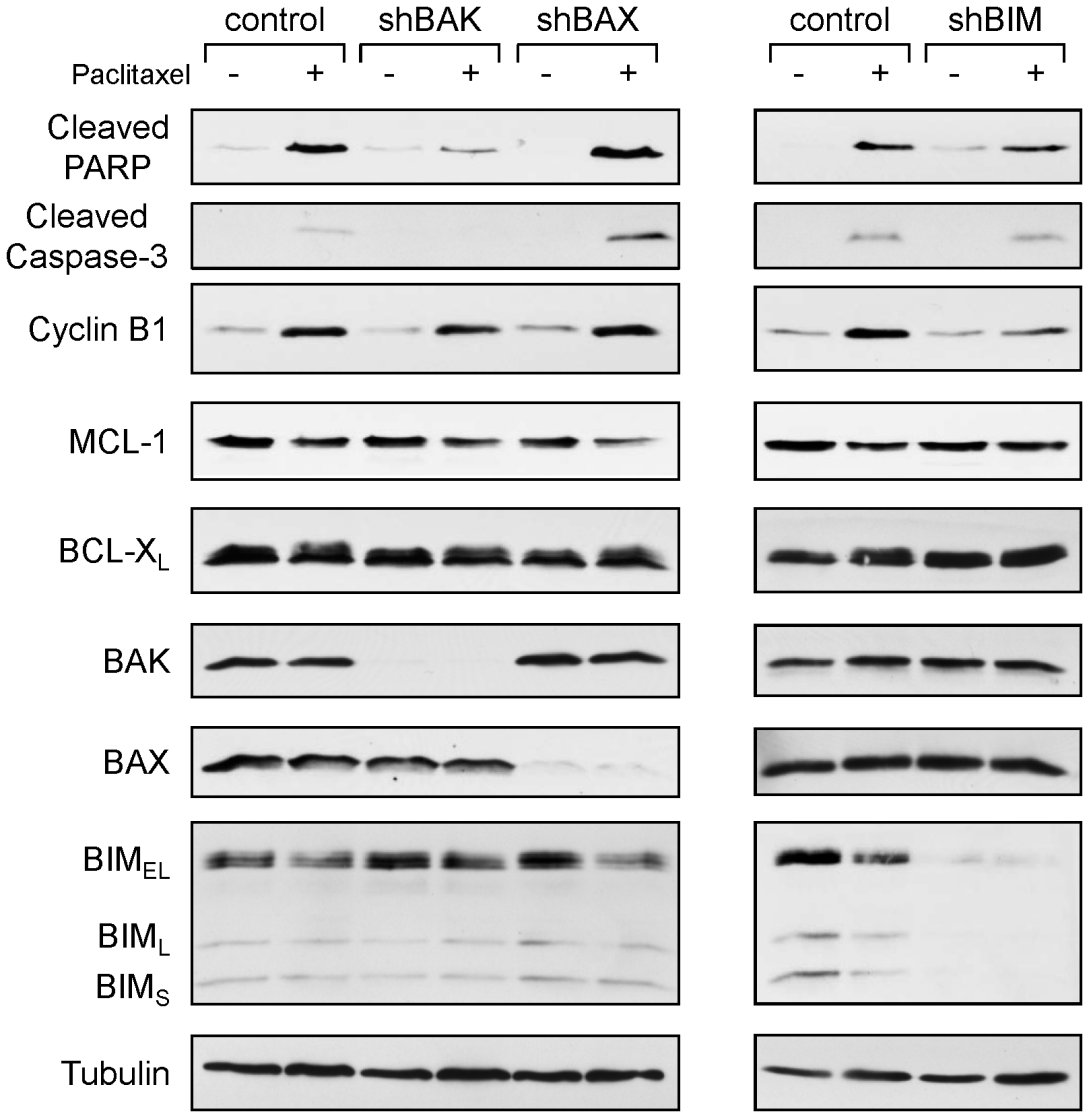
BAK, but neither BAX nor BIM, plays a role in paclitaxel-induced apoptosis in human breast cancer cells. SK-BR-3 cells were infected with lentiviruses expressing shRNAs for non-targeting control, BAK, BAX, or BIM. Puromycin-resistant cells were pooled after each infection. Cells were treated with 20 nM paclitaxel for 24 hours and equal amounts of total cell extracts were subjected to Western blotting with the indicated antibodies.

In order to examine the role of BAK in paclitaxel-sensitivity in human breast cancer cell lines, we determined the levels of BAK in five human breast cancer cell lines. Among the cell lines we examined, SK-BR-3 showed the highest and T47-D showed the lowest BAK expression (Figure S1A). In accordance with BAK expression, SK-BR-3 was more sensitive to paclitaxel than T47D, implicating that the level of BAK could determine the paclitaxel sensitivity (Figure S1B). We then examined whether BAK played a role in paclitaxel-induced apoptosis. Down-regulation of BAK by shRNA in both SK-BR-3 and T47-D inhibited PARP cleavage, indicative of apoptosis (Figure 5). The levels of MCL-1 were gradually decreased with paclitaxel treatment in both control cells and the cells in which BAK was down-regulated by shRNA. We further established SK-BR-3 cells in which BAX or BIM was down-regulated by shRNAs (Figure 6). In contrast with BAK down-regulation, BAX or BIM down-regulation did not change paclitaxel sensitivity. Similar results were also observed in MDA-MB-468 cells in which BAK or BIM was down-regulated by shRNA (Figure S2). Treatment with paclitaxel induced Cyclin B1, indicative of G2/M arrest, and the decrease of MCL-1 in control and all shRNA-transfected SK-BR-3 cells (Figure 6), suggesting that the decrease of MCL-1 is not the consequence of cell death [19]. Taken together, BAK plays a critical role in paclitaxel-induced apoptosis in human breast tumor cells.

### BAK is Activated with Paclitaxel Treatment by the Release from the BAK/MCL-1 Complex

**Figure 7:**
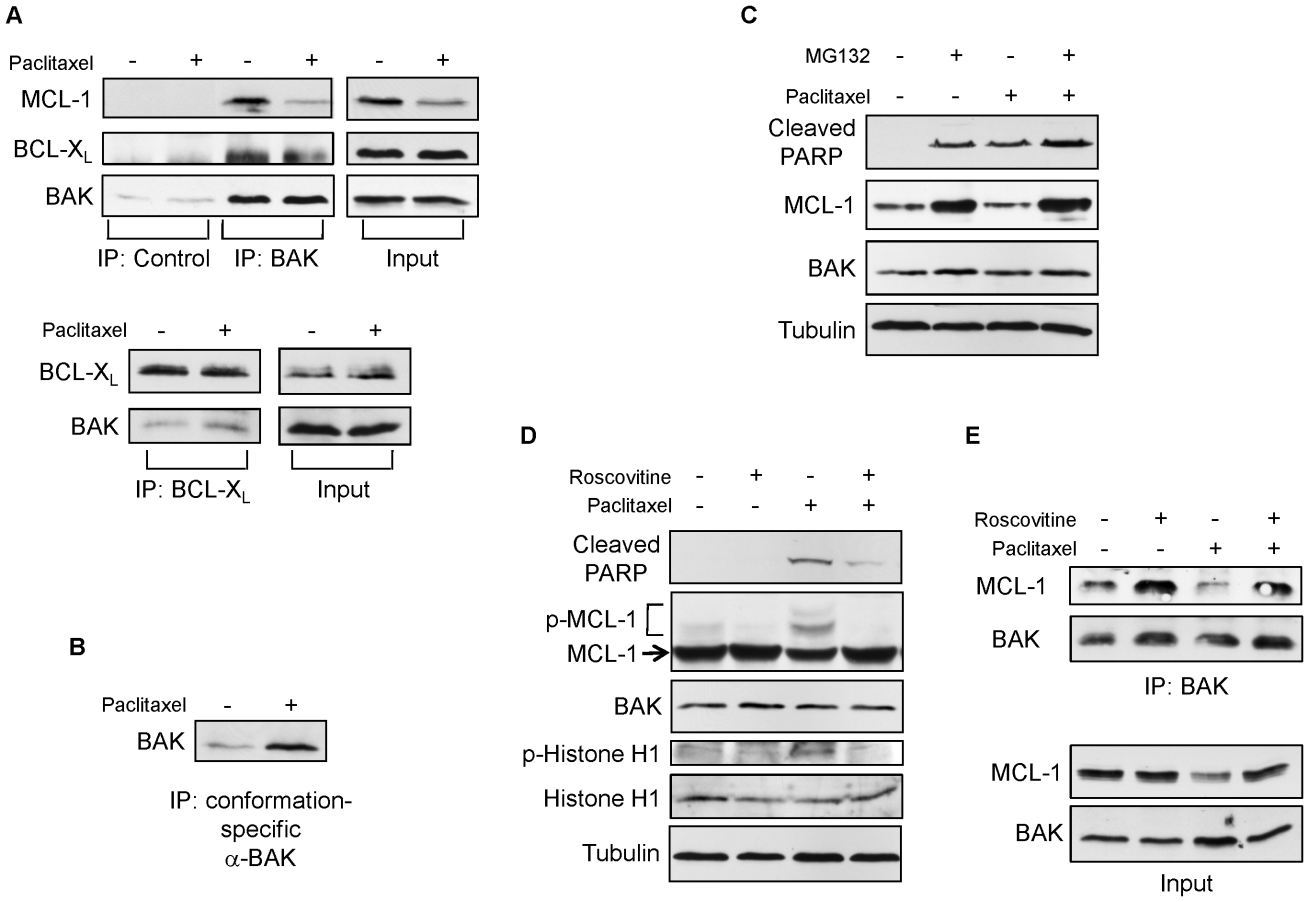
BAK is activated with paclitaxel treatment by the release from the BAK/MCL-1 complex. (A) SK-BR-3 cells were treated with 20 nM paclitaxel for 24 hours. Immunoprecipitations with the total cell extracts were carried out with an anti-BAK, rabbit IgG (Control) (Upper panel) or an anti-BCL-X_L_ antibody (Lower panel). Western blotting was carried out on precipitated samples with the indicated antibodies. (B) The SK-BR-3 total cell extracts in (A) were subjected to immunoprecipitations with a BAK (Ab-1) conformational change-specific antibody. Western blotting was carried out on precipitated samples with an anti-BAK antibody. (C) SK-BR-3 cells were pre-treated with 5 µM MG132 for 30 minutes, and were then treated with 20 nM paclitaxel for 24 hours. Total cell extracts were subjected to Western blotting with the indicated antibodies. (D) SK-BR-3 cells were pre-treated with 10 µM roscovitine for 30 minutes, and were then treated with 20 nM paclitaxel for 24 hours. Total cell extracts were subjected to Western blotting with the indicated antibodies. (E) Immunoprecipitations with the total cell extracts from (D) were carried out with an anti-BAK antibody. Western blotting was carried out on precipitated samples with the indicated antibodies.

BAK is known to bind MCL-1 and BCL-X_L_, the anti-apoptotic BCL-2 family proteins, before cell death signals are transduced. Upon death stimuli, BAK is released and is activated to induce downstream cell death pathways [20]. Immunoprecipitation experiments with SK-BR-3 confirmed that BAK interacted with MCL-1 and BCL-X_L_ before paclitaxel treatment (Figure 7A). After the treatment, the level of BAK was unchanged and the level of MCL-1 was decreased. Accordingly, BAK/MCL-1 interaction was decreased (Figure 7A, upper panel). In contrast, the level of BCL-X_L_ and BAK/BCL-X_L_ interaction were not altered (Figure 7A, upper panel). A reciprocal immunoprecipitation experiment with the BCL-X_L_ antibody confirmed BAK/BCL-X_L_ interaction was not altered (Figure 7A, lower panel). Decrease of BAK/MCL-1 interaction led to the activation of BAK, judged by the increase of BAK conformational change (Figure 7B). These results indicate that BAK is released from the BAK/MCL-1 complex with paclitaxel treatment by the decrease of MCL-1 protein level, followed by the activation of BAK and apoptosis.

It has been shown that the degradation of MCL-1 with microtubule damaging agents such as paclitaxel and vinblastine is mediated through its phosphorylation by Cdk1 followed by proteasome-dependent degradation [21], [22]. When SK-BR-3 cells were co-treated with paclitaxel and a proteosome inhibitor MG132, MCL-1 expression was increased (Figure 7C). MCL-1 expression was also increased in cells treated with MG132 alone, consistent with reports showing that it is normally subject to rapid turnover via proteosome-mediated degradation in untreated cells [23]. With the condition we used, treatment of MG132 for 24 hrs by itself induced apoptosis, and combination with paclitaxel and MG132 increased apoptosis presumably through the MCL-1-independent mechanisms. It has also been reported that the combination with paclitaxel and MG132 synergistically induces apoptosis in breast cancer cells [24], [25]. Our result suggests that loss of MCL-1 is due to proteosome-mediated degradation. The Cdk inhibitor roscovitine blocked paclitaxel-induced histone H1 phosphorylation (a substrate for Cdk1), MCL-1 phosphorylation, and the decrease of intact MCL-1 protein (Figure 7D). This result suggests that Cdk1 mediated phosphorylation of MCL-1 triggers the degradation. As a consequence of inhibition of MCL-1 degradation, more BAK/MCL-1 complex was detected by co-immunoprecipitation with anti-BAK, and cells showed less apoptosis induced with paclitaxel treatment judged by the amount of cleaved PARP (Figure 7D and 7E). The inhibition of MCL-1 degradation by MG132 or roscovitine was also observed in MDA-MB468 or T47-D cells (Figure S3). Taken together, the results indicate that BAK is activated with paclitaxel treatment by the release from the BAK/MCL-1 complex via Cdk1-mediated phosphorylation of MCL-1 followed by proteasome-dependent MCL-1 degradation.

## Discussion

Paclitaxel, the prototypic member of taxanes, is used in the treatment of breast, ovarian, and lung cancers [1], [2]. This chemotherapeutic drug causes mitotic arrest, however precisely how this mitotic arrest triggers subsequent cell death (apoptosis) is still unclear. Several reports have demonstrated a paclitaxel-mediated dependence on BIM, a BH3-only pro-apoptotic BCL-2 family protein [5], [6], [7], [8], [9]. On the other hand, it has been recently reported that depletion of BIM does not impart paclitaxel resistance to HeLa cells or breast carcinoma cell lines MCF-7, SK-BR-3, and MDA-MB-468 [10]. Thus, more studies need to be undertaken in order to reconcile the previously characterized role of BIM in paclitaxel-induced cell death in mouse models and in clinically relevant cells such as human breast cancer cells.

We therefore examined the role of BIM in paclitaxel-induced cell death in a variety of experimental systems in *in vitro* and *in vivo*; *bim^−/−^* MEFs (Figure 1), the *bim^−/−^* mouse breast tumor model (Figure 2 and 3), and shRNA-mediated down-regulation of BIM in human breast cancer cells (Figure 6). In the systems we have examined, we conclude that BIM is dispensable in apoptosis with paclitaxel treatment. At this moment, the reasons of the discrepancy are unclear between our results and previous observations from others. Of note, the concentrations of paclitaxel used in Czernick *et al.* [10] and Zhou *et al.* [26] are relatively low (20–50 nM). We carefully determined the concentrations used to examine minimal toxicity for each cell type (20 nM for SK-BR-3 and MDA-MB-468; 50 nM for MEFs and T47D). It is well known that paclitaxel exerts its mitotic effects by alternate mechanisms, depending on the concentration of the drug utilized [1]. Thus, it is possible that the mechanisms of paclitaxel-induced cell death are also concentration-dependent.

*Bim*-deficient tumors grew slower than the wild-type tumors (Figure 2), suggesting that BIM plays a role in ErbB2-mediated tumor growth. It has been reported that BIM deficiency promotes the development of B cell lymphomas in Eµ-*myc* transgenic mice and tumorigenesis of E1A and dominant-negative p53 transformed BMK (baby mouse kidney) cells in nude mice [9], [27]. Therefore, the role of BIM in tumorigenesis may be context-dependent. One different point between our study and others could be p53-dependency in tumor development. Whereas the p19Arf/p53 pathway is frequently mutated in tumors arising in *Bim* ^+*/*+^ Eµ-*myc* mice, it is unaffected in most *Bim*-deficient tumors, indicating that BIM reduction is an effective alternative to loss of p53 function [27]. Since the BMK cells are transformed with genes that disrupt the RB and p53 pathways, BIM controls a checkpoint independent of those major routes to tumorigenesis [9]. In contrast, ErbB2-mediated tumorigenesis is controlled by cell cycle regulators including p53 [28]. Obviously, further studies are required to clarify these issues.

Since *bax^−/−/^bak^−/−^* MEFs are insensitive to paclitaxel treatment, we examined the possible independent role of BAX or BAK. Not only *bak^−/−^* MEFs, but also down-regulation of BAK in several human breast cancer cells showed paclitaxel resistance (Figure 4, 5, 6, and S2). Consistent with our observation, it has been shown that down-regulation of BAK in MDA-MB-435 and MDA-MB-231 human breast cancer cell lines suppress paclitaxel-induced apoptosis [26]. In contrast, *bax^−/−^* MEFs or down-regulation of BAX in breast cancer cells showed similar paclitaxel sensitivity as wild-type MEF or control cells, respectively (Figure 4 and 6). The roles of BAX and BAK can be redundant or non-redundant, depending on the apoptotic stimuli. BAK plays an essential role for apoptosis induced by Semliki Forest virus, gliotoxin, BCL-X_S_, inhibitors of protein synthesis (*Pseudomonas* exotoxin A, cycloheximide, ricin), and vinblastine [22], [29], [30], [31], [32]. Among them, vinblastine belongs to mictotubule damaging agents as paclitaxel, thus these agents could induce a common mechanism(s) to execute apoptosis. We examined BAX conformational change by a conformation specific antibody (6A7). We found that BAX conformation was also changed by paclitaxel treatment (data not shown). However, our data clearly demonstrate that paclitaxel-induced apoptosis is BAX-independent. There are several controversial reports about the role of BAX in paclitaxel or vinblastine treatment [33], [34], [35]. Thus, more study will be required to determine the significance of BAX conformational change with paclitaxel treatment.

Our work defines a rational mechanism whereby loss of MCL-1 leads to apoptosis in human cells. We showed that in untreated cells BAK was bound to MCL-1. BAK/MCL-1 interaction was decreased and BAK was released with paclitaxel treatment in concert with loss of MCL-1 expression. BAK underwent conformational changes recognized by an active BAK antibody in response to paclitaxel treatment. Co-treatment with paclitaxel and MG132 inhibited the decrease of MCL-1 expression. Thus, proteasome-dependent MCL-1 degradation during mitotic arrest leads to loss of sequestration of BAK permitting BAK activation and apoptosis. We performed knockdown of MCL-1 expression in SK-BR3 and MDA-MB468 cells. Both cell lines started to die within 24 hours without any treatment. The induction of apoptosis by knockdown of MCL-1 expression in SK-BR-3 cells has been previously reported [36]. These observations are consistent with our idea that MCL-1 degradation during mitotic arrest leads to loss of sequestration of BAK permitting BAK activation and apoptosis. In contrast, the level of BCL-X_L_ was not changed and BAK/BCL-X_L_ interaction was not altered. Our study indicates that MCL-1 degradation may elicit apoptosis after paclitaxel treatment via BAK released from suppressed complexes, whereas BCL-X_L_ is unaffected. In contrast to the observation in human breast tumor cells, we could not detect MCL-1 degradation upon paclitaxel treatment in MEFs and mouse mammary epithelial cells. Human and mouse MCL-1 are highly conserved although several stretches of amino acids are diverged (Figure S4). Treatment with roscovitine prevented paclitaxel-induced MCL-1 degradation, suggesting the contribution of Cdk1. Putative Cdk1 phosphorylation sites in human MCL-1, Ser64 and Thr92 [37], [38] do not exist in mouse MCL-1. Thus, the mechanisms of BAK activation with paclitaxel treatment might be different in between human and mouse cells.

We show that BAK plays an important role in paclitaxel sensitivity of breast cancer cells. BAK can be a prognostic marker to determine paclitaxel sensitivity of breast cancer patients. BAK may potentially serve as a therapeutic target for overcoming paclitaxel resistance in human breast cancer. These novel findings have important implications in the development of targeted therapeutics for overcoming paclitaxel resistance in human breast cancer.

## Supporting Information

Figure S1 **The level of BAK expression and paclitaxel sensitivity in human breast cancer cells.** (A) Total cell extracts of the indicated human breast cancer cells were subjected to Western blotting with BAK or tubulin antibodies. (B) SK-BR-3 cells and T47-D cells were treated with the indicated concentrations of paclitaxel for 48 hours. Cell death was determined by trypan-blue exclusion. Average values from triplicate samples are shown.
(TIF)

Figure S2 **BAK, but not BIM, plays a role in paclitaxel-induced apoptosis in human breast cancer cells.** MDA-MB-468 cells were infected with lentiviruses expressing shRNAs for non-targeting control, BAK or BIM. Puromycin-resistant cells were pooled after each infection. Cells were treated with 20 nM paclitaxel for 24 hours and equal amounts of total cell extracts were subjected to Western blotting with the indicated antibodies.
(TIF)

Figure S3 **Paclitaxel-induced MCL-1 degradation was blocked by a proteasome inhibitor MG132 or a Cdk inhibitor roscovitine in MDA-MB468 and T47-D cells.** (A) MDA-MB468 cells were pre-treated with 5 µM MG132 for 30 minutes, and were then treated with 20 nM paclitaxel for 24 hours. Total cell extracts were subjected to Western blotting with the indicated antibodies. (B) MDA-MB468 and T47-D cells were pre-treated with 10 µM roscovitine for 30 minutes. MDA-MB468 cells were then treated with 20 nM paclitaxel for 24 hours and T47-D cells were treated with 50 nM paclitaxel for 48 hours. Total cell extracts were subjected to Western blotting with the indicated antibodies.
(TIF)

Figure S4 **Comparison of amino acid sequences between human and mouse MCL-1.** Alignment of the primary amino acids sequence of human and mouse MCL-1 is shown. The middle line indicates the same amino acids between the species.+indicates an amino acid similarity. Ser64 and Thr92 in human MCL-1 are indicated with bold-italic.
(TIFF)
