# Supplementary figures and images for: Paclitaxel-Induced Apoptosis Is BAK-Dependent, but BAX and BIM-Independent in Breast Tumor
Source: PLoS One. 2013 Apr 5;8(4):e60685. doi: 10.1371/journal.pone.0060685 (PMC3618047; doi:10.1371/journal.pone.0060685)

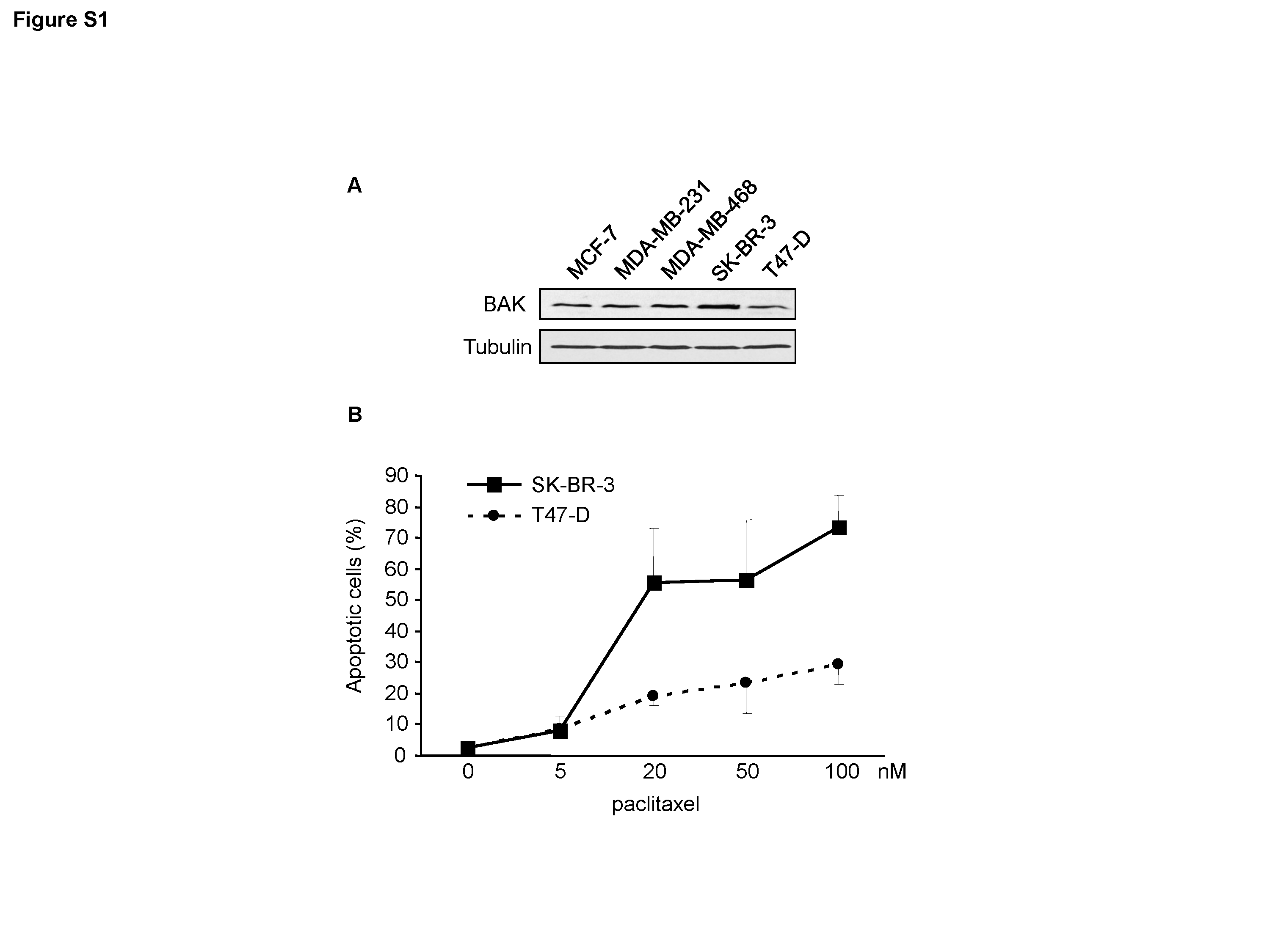

Supplement: Figure S1 — The level of BAK expression and paclitaxel sensitivity in human breast cancer cells. (A) Total cell extracts of the indicated human breast cancer cells were subjected to Western blotting with BAK or tubulin antibodies. (B) SK-BR-3 cells and T47-D cells were treated with the indicated concentrations of paclitaxel for 48 hours. Cell death was determined by trypan-blue exclusion. Average values from triplicate samples are shown. (TIF) [file pone.0060685.s001.tif]

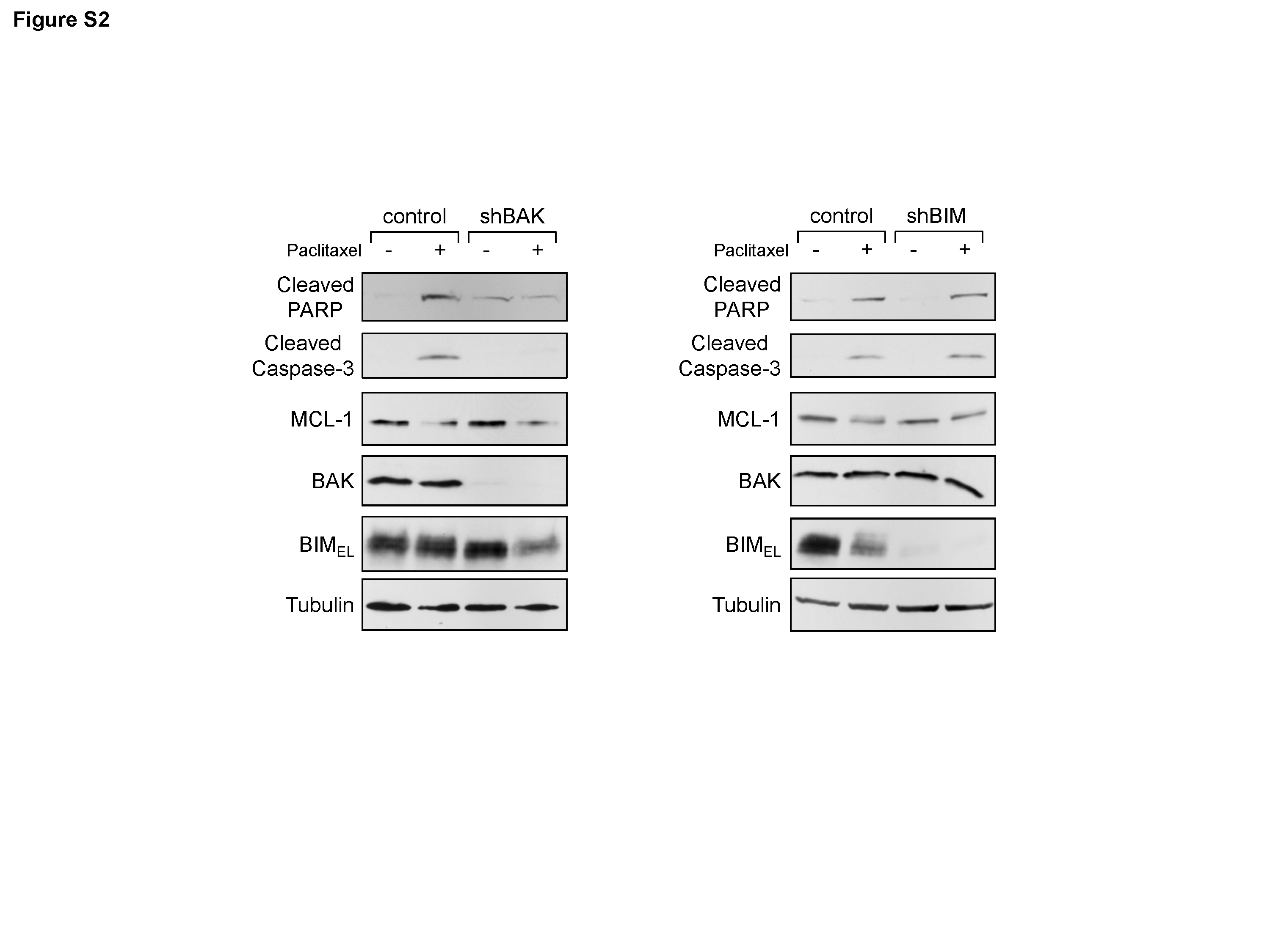

Supplement: Figure S2 — BAK, but not BIM, plays a role in paclitaxel-induced apoptosis in human breast cancer cells. MDA-MB-468 cells were infected with lentiviruses expressing shRNAs for non-targeting control, BAK or BIM. Puromycin-resistant cells were pooled after each infection. Cells were treated with 20 nM paclitaxel for 24 hours and equal amounts of total cell extracts were subjected to Western blotting with the indicated antibodies. (TIF) [file pone.0060685.s002.tif]

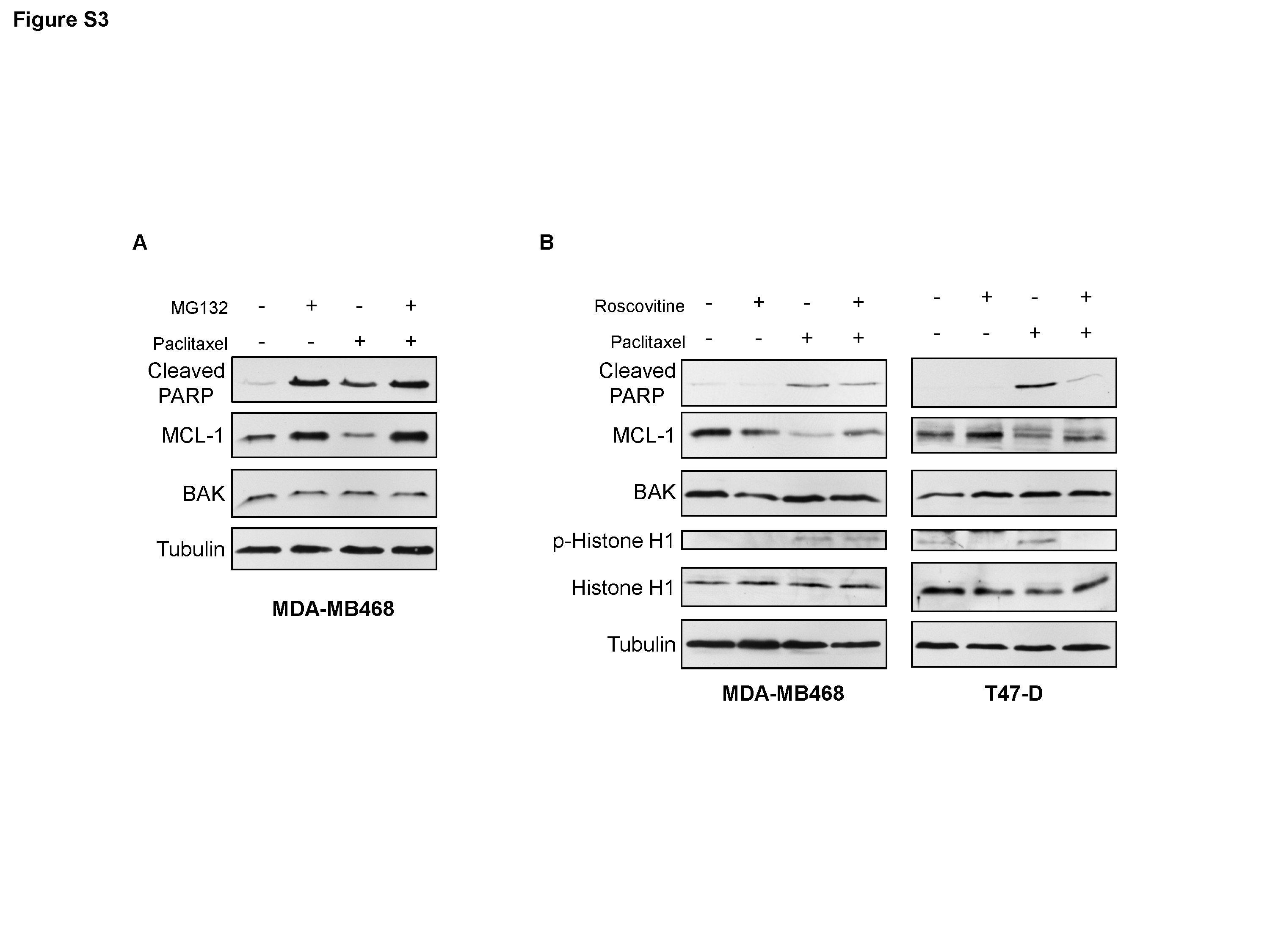

Supplement: Figure S3 — Paclitaxel-induced MCL-1 degradation was blocked by a proteasome inhibitor MG132 or a Cdk inhibitor roscovitine in MDA-MB468 and T47-D cells. (A) MDA-MB468 cells were pre-treated with 5 µM MG132 for 30 minutes, and were then treated with 20 nM paclitaxel for 24 hours. Total cell extracts were subjected to Western blotting with the indicated antibodies. (B) MDA-MB468 and T47-D cells were pre-treated with 10 µM roscovitine for 30 minutes. MDA-MB468 cells were then treated with 20 nM paclitaxel for 24 hours and T47-D cells were treated with 50 nM paclitaxel for 48 hours. Total cell extracts were subjected to Western blotting with the indicated antibodies. (TIF) [file pone.0060685.s003.tif]
